# Understanding Clinician Macrocognition to Inform the Design of a Congenital Heart Disease Clinical Decision Support System

**DOI:** 10.3389/fcvm.2022.767378

**Published:** 2022-02-03

**Authors:** Azadeh Assadi, Peter C. Laussen, Gabrielle Freire, Patricia Trbovich

**Affiliations:** ^1^Department of Critical Care Medicine, Labatt Family Heart Centre, Toronto, ON, Canada; ^2^Department of Engineering and Applied Sciences, Institute of Biomedical Engineering, University of Toronto, Toronto, ON, Canada; ^3^Institute of Medical Sciences, University of Toronto, Toronto, ON, Canada; ^4^Executive Vice President for Health Affairs, Boston Children's Hospital, Boston, MA, United States; ^5^Harvard Medical School, Boston, MA, United States; ^6^Division of Emergency Medicine, Department of Pediatrics, University of Toronto, Toronto, ON, Canada; ^7^Institute of Health Policy Management and Evaluation, University of Toronto, Toronto, ON, Canada; ^8^Research and Innovation, North York General Hospital, Toronto, ON, Canada

**Keywords:** decision support, congenital heart disease - cardiac, macrocognition, cognitive task analysis, digital health (eHealth), emergency medicine (MeSH database)

## Abstract

**Background and Objectives:**

Children with congenital heart disease (CHD) are at risk of deterioration in the face of common childhood illnesses, and their resuscitation and acute treatment requires guidance of CHD experts. Many children with CHD, however, present to their local emergency departments (ED) with gastrointestinal and respiratory symptoms that closely mimic symptoms of CHD related heart failure. This can lead to incorrect or delayed diagnosis and treatment where CHD expertise is limited. An understanding of the differences in cognitive decision-making processes between CHD experts and ED physicians can inform how best to support ED physicians when treating CHD patients.

**Methods:**

Cardiac intensivists (CHD experts) and pediatric emergency department physicians (ED physicians) in a major academic cardiac center were interviewed using the critical decision method. Interview transcripts were coded deductively based on Schubert and Klein's macrocognitive frameworks and inductively to allow for new or modified characterization of dimensions.

**Results:**

In total, 6 CHD experts and 7 ED physicians were interviewed for this study. Although both CHD experts and ED physicians spent a lot of time sensemaking, their approaches to sensemaking differed. CHD experts reported readily recognizing the physiology of complex congenital heart disease and focused primarily on ruling out cardiac causes for the presenting illness. ED physicians reported a delay in attributing the signs and symptoms of the presenting illness to congenital heart disease, because these clinical findings were often non-specific, and thus explored different diagnoses. CHD experts moved quickly to treatment and more time anticipating potential problems and making specific contingency plans, while ED physicians spent more time gathering a range of data prior to arriving at a diagnosis. These findings were then applied to develop a prototype web-based decision support application for patients with CHD.

**Conclusion:**

There are differences in the cognitive processes used by CHD experts and ED physicians when managing CHD patients. An understanding of differences in the cognitive processes used by CHD experts and ED physicians can inform the development of potential interventions, such as clinical decision support systems and training pathways, to support decision making pertaining to the acute treatment of pediatric CHD patients.

## Introduction

Children born with anatomic defects of their heart, called congenital heart disease (CHD), can have a wide range of defects that affect how blood flows through the heart and lungs and to the body. Treatment has substantially improved over the past 4 decades, with heart surgery frequently undertaken in the newborn period or early infancy to correct defects. Despite this, a residual burden of disease following their corrective interventions can predispose patients with CHD to a high risk of deterioration from common childhood illnesses and can hinder their responses to traditional resuscitation efforts (1). When acutely ill, many patients present to their community emergency departments (ED) where CHD expertise is limited, as emergency medicine physicians are not specifically trained in the acute treatment of CHD. These children often present with gastrointestinal and respiratory illnesses which often mimic symptoms of heart failure (2–6). This could lead to delays and errors in diagnosis and care in the absence of an assessment by clinicians with CHD expertise. It is not known whether CHD experts and ED physicians apply different cognitive processes to understand and treat acutely ill pediatric CHD patients. An understanding of these differences can be used to augment training and develop interventions to help clinicians better recognize cues and patterns of acutely ill pediatric CHD patients presenting to EDs.

CHD is the most common congenital condition in newborns (7). With the advances in diagnostic, surgical, medical, and interventional therapies in the field, many of these children are surviving to adulthood after their initial hospitalization and corrective interventions, achieving an acceptable quality of life, and are living in diverse communities. It is projected that in the next 5–10 years, the number of adults living with CHD will be more than the number of children born with it each year (8). The burden of CHD is such that complications can develop overtime and regular longer term follow up by experts trained in CHD is important. However, given the diverse and dispersed communities in which patients with CHD live, immediate access to care by CHD experts is not guaranteed. During acute illness, patients with CHD may present to emergency department or primary care physician offices and require urgent care by clinicians who are not trained in CHD. CHD patients represented 0.17% of 241 million ED visits recorded in the Nationwide Emergency Department Sample (NEDS) between 2006 and 2014 (6). The majority of these patients were under 1 year of age and were more likely to die, require hospital admission, or transfer to specialty centers (6).

A survey of ED physicians in the state of Michigan reveals an overall lack of comfort among ED physicians when asked to care for acutely ill pediatric patients with single ventricle physiology [a severe form of CHD where blood from the body and the lungs mixes in the heart such that the oxygen level (saturation) in the blood is lower (usually <85%) when compared to a patient with two normal ventricles where blood from the heart and lungs are separated and oxygen saturations are >95%] (9). When asked about the expected arterial oxygen saturation for these patients, 52% of general ED physicians and 35% of pediatric ED physicians were unsure of their response (9). Moreover, 18% of general ED physicians and 26% of pediatric ED physicians expressed an incorrect saturation expectation of these patients (9). In addition to a lack of specific training in acute CHD, other reasons for this lack of overall comfort and familiarity with CHD patients include the lack of detailed information about the CHD in a particular patient, the unique and complex medical language used by experts to describe the myriad of possible CHDs (10, 11) and limitations in access to in-house CHD experts (9).

Clinical decision making, particularly under the conditions of critical illness in the ED or the intensive care unit (ICU), is complex. Proposing solutions to augment care for acutely ill pediatric CHD patients in the ED, requires an understanding of the clinicians' cognition to help clinical decision making. Macrocognition is the study of cognitive processes (i.e., mental functions involved in the acquisition, storage, interpretation, manipulation, and use of knowledge) used to perform a task under complex conditions where there is uncertainty and time pressure (12, 13). Methodologies of macrocognition focus on understanding decision making in realistic scenarios and environments rather than laboratory settings (13). Cognitive Task Analysis (CTA) is one of such methodologies that has been used to elicit and understanding of macrocognitive processes involved in performing various clinical tasks (14–17). It can be defined as a set of methods to elicit, explain, and represent the mental processes involved in performing a task (18). Such techniques have previously been used to improve clinicians' access to data through electronic platforms (16), develop and integrate decision support systems (CDSS) into the clinical space (16, 19–21), develop simulation training programs to aid in sepsis recognition (22), and to understand decision making among expert clinicians (15, 17). The goal of this study is to understand how CHD experts and ED physicians (as non-experts in CHD) make clinical decisions regarding the acute care of pediatric CHD patients. The similarities and differences in the macrocognition of the two, including the types and ways in which data are used to arrive at diagnoses and treatment decisions, can inform clinician training and the development of clinical decision supports to address gaps in knowledge and/or practice.

## Materials and Methods

Institutional Research Ethics Board approval was obtained for this study prior to recruitment (REB#1000064567). Further methodological details to what is described here can be found in our published protocol (23). To explore the differences in the cognitive processes (i.e., the knowledge, skills, and strategies) used by CHD experts compared to ED physicians when treating CHD patients, semi-structured interviews were conducted using the Critical Decision Method (CDM). CDM is a specific type of CTA. The CDM is a semi-structured interview technique to study challenging incidents, by eliciting concrete assessment indicators (e.g., cues and patterns) to treat incidents, particularly those that might have been missed by less experienced personnel (24). Specifically, critical decision method requires participants to retrospectively recount events from their perspective to elicit knowledge from working in challenging and atypical complex situations (25, 26).

Probing questions used for this study were adapted from Baxter et al. (19), and Schubert et al. (15) to focus on differentiating between the macrocognitive processes of CHD experts and ED physicians caring for acutely ill pediatric CHD patients. The questions were refined by AA and PT from a human factors engineering and CTA perspective, AA and PL from a CHD expertise perspective, and GF from an emergency medicine perspective. Once questions were validated by the investigators, a pilot interview was done to ensure consistency of questions and adequacy of time allotted for the study. The pilot interview was then transcribed and analyzed using the same macrocognitive framework proposed for the study with good results. No changes were deemed necessary to the probing questions or timing of the interview and the study was proceeded as proposed.

### Setting and Participants

Until reaching data saturation within each group, pediatric CHD experts and ED physicians trained in acute pediatric treatment and resuscitation were recruited from a large pediatric academic center with university affiliated training programs for both the Pediatric Cardiac Intensive Care Unit (PCICU) and pediatric ED. This was also a major cardiac specialty center with hundreds of complex pediatric CHD surgeries each year. The PCICU at this hospital cares for ~800 pediatric patients with CHD while the ED sees over 50,000 pediatric patients with 6,000 admissions annually (27, 28). CHD experts were selected from the 9 pediatric cardiac intensivists of this PCICU, all of whom had at least 3 years of specialized training in pediatric CHD and at least 1 year experience as a consultant in the acute treatment of pediatric CHD. ED physicians were selected from the pediatric emergency physicians without any formal training in acute pediatric CHD treatment and at least 1 year experience after completing training. Of these ED physicians, 12 could recall caring for a pediatric CHD patient presenting with respiratory or GI symptoms. Rolling recruitment was email based and continued until saturation was reached, defined as the point where no new themes emerged.

### Interview and Data Analysis

Semi-structured interviews were conducted one-on-one with participants on a virtual conferencing platform. Participants were free to choose a location of their convenience for the virtual interview conducted by AA, a pediatric nurse practitioner in the cardiac critical care unit at the study institution and a doctoral student in human factors engineering. During these 2-h interviews, participants were asked to recall a clinical scenario from their experience caring for an acutely ill pediatric CHD patient and answer questions about their decisions, the data they used, their understanding of the scenario, and their overall thoughts and logic as they treated the patient. Individual comfort in managing patients with single ventricle defects as the most severe form of CHD was asked of all participants. Interviews were audio recorded, transcribed verbatim, and analyzed using NVivo 12^®^ software. For member checking, each participant was then asked to review a summary of their interview to check for possible omission and misinterpretation. Two coders (AA and MD) who both had extensive experience coding interview data independently analyzed interview transcripts deductively based on a priori macrocognitive processes identified from Klein's original macrocognition framework (25) and Schubert's (15) subsequent modifications to that framework, as well as inductively to allow for the flexibility of uncovering new processes. Coders reviewed discrepancies until consensus was reached and the set of agreed on macrocognitive processes comprised the analytical framework that was used to independently code the interviews. A Cohen's Kappa of 0.84 was achieved through iterative review between both coders, after which, the remainder of the transcripts were coded by one coder. The detailed methodology for this study has been previously published (23).

## Results

For this study, of the 9 available CHD experts, 67% (*n* = 6) and 58% (*n* = 7) of 12 ED physicians who met the inclusion criteria were interviewed. Saturation was reached at *n* = 5 and *n* = 6, respectively. All participants completed the entirety of the interview. Table 1 shows demographic characteristics of the participants in each group.

**Table 1:** Participant characteristics.

		**CHD experts**	**ED physicians**
Participants' gender	Female	3	4
	Male	3	3
Experience within their specialty	More than 15 yrs	1	0
	5–15 yrs	2	5
	<5 yrs	3	2
Experience with acute CHD treatment	More than 30 patients/yr	6	3
	10–30 patients/yr	0	2
	<10 patients/yr	0	2
Comfort treating a pediatric single ventricle without in-house pediatric cardiology	Very comfortable	3	0
	Comfortable	3	1
	Somewhat uncomfortable	0	5
	Uncomfortable	0	1
	Worried	0	0

### Macrocognitive Processes of CHD-Experts and ED Physicians When Treating Acutely Ill Pediatric CHD Patients

**Figure 1:**
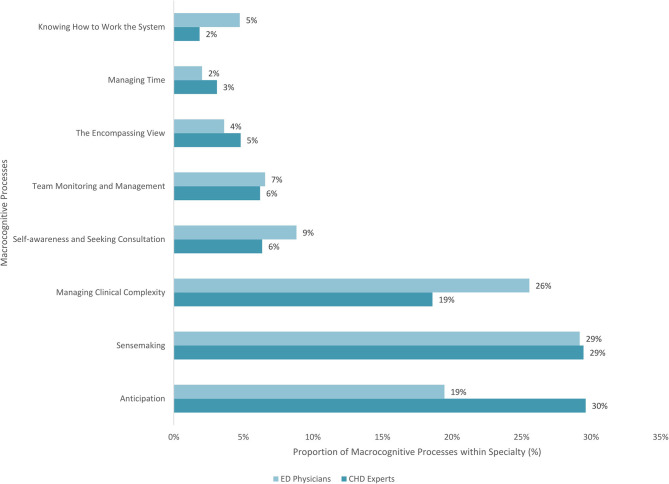
Distribution by number of verbal references and percentages of macrocognitive processes for CHD experts and ED physicians.

There were 1,087 verbal references related to 8 macrocognitive dimensions across both CHD experts and ED physicians. Figure 1 displays the differences between CHD experts and ED physicians for each cognitive dimension. The highest number of verbal references for CHD experts was related to *anticipation* and *sensemaking*, accounting for a total of 59% of their verbal references. For ED physicians, the highest number of verbal references was to *sensemaking* and *managing clinical complexity*, accounting for a total of 55% of their verbal references. CHD experts moved to diagnosis quickly because they recognized the physiology of a CHD, and consequently spent more time anticipating how a situation would unfold and mitigating potential problems. Whereas ED physicians kept an open framework to allow room for a broader differential set of diagnoses (e.g., sepsis, infection), and consequently spent time gathering a wide range of data and gradually initiating therapies to what they interpreted. While both CHD experts and ED physicians spent a major proportion of their time sensemaking, there were notable differences in the way they did so. Experts reported that they quickly considered details of patients' CHD anatomy, and thoroughly evaluated if patients' unique cardiac anatomy could cause the presenting symptoms. By contrast, ED physicians considered general presenting symptoms and developed several differential diagnoses, without narrowing in on cardiac causes on first encounter. Table 2 provides further differentiation of how cognitive processes were expressed by experts and ED physicians.

**Table 2:** Differences between macrocognitive processes of CHD experts and ED physicians when managing pediatric CHD.

**Macrocognitive processes**	**CHD experts**	**ED physicians**
The encompassing view	Describe patient with their overall physiological state and rapidly focus on cardiac issues and symptoms. *Example: “Poor pulmonary blood flow” (EXP02)*	Describe the patient in terms of overall acuity and the organ system most effected, always maintaining a broad list of differentials. *Example: “Respiratory failure” (PEM07)*
Sense-making	Recognize trends and patterns unique to each CHD and quickly focus on ruling out cardiac etiologies. *Example: “If you just tell me 14 year old Tetralogy of Fallot repair, there's an expectation of where things are at. So I know what the anatomy is, … what the variability of the anatomy could be, … what the repair is. I understand the variations in … what residual lesions could be or the long-term complications of it. So does she have stenosis? … regurgitation? … RV dysfunction? … RV [dilation]? What's her QRS? Is it prolonged? Is she at risk for ventricular arrhythmias because of the prolonged QT prolonged QRS. You know, all those things, I think automatically now come to my brain.” (EXP06)*	Do not narrow in on CHD specific patterns and cues as readily as CHD experts. Keep an open framework and broad list of possible differentials. *Example: “there's so much that can go wrong with the [heart] in addition to all the other standard stuff that you worry about that time of year, like pneumonia, sepsis, and endocrine things and stuff like that. … I found out [from cardiology] that this kid had a fenestrated Fontan and [cardiology mentioned that] sometimes we worry they can get clotted and that's why they dropped their saturations and [so then] I asked, all right, what do I do with that information?” (PEM06)*
Anticipation	Anticipate course and potential interventions based on known trajectory of each CHD. *Example: “Reviewed saturation goals and that if the baby needed positive pressure, it would be okay given the transition period” (EXP05)*	Anticipate potential for deterioration but not the CHD specific trajectory or mechanism of deterioration. *Example: “Made sure we had resuscitation cart outside the room and the drug doses for resuscitation made readily available.” (PEM05)*
Managing clinical complexity	Prioritize targeted tests but gather a wide range of data and often anticipate results. Initiate therapies in parallel to achieve hemodynamic stability quickly. *Example: “…talking more to the ECHO people about, can you identify which ventricle is more dominant? And how restrictive is the VSD?” (EXP05)*	Obtain a wide range of non-CHD specific tests while maintaining an open framework and gradually initiating broad therapy to restore baseline. *Example: “Thought to get a blood gas, to see what her acid-base state was and some other basic things like, a CBC and electrolytes for good measure” (PEM01)*
Knowing how to work the system	Know how to effectively consult further expertise as needed. Able to communicate their specific cardiac concerns and questions clearly. *Example: “Asked the catheterizers how concerned they were about potential distal embolization when they intervened on the clot” (EXP03)*	Express difficulties at times communicating their concerns and the right data with the CHD experts. *Example: “it is really hard to find the information [in] easily accessible manner. … even just decoding exactly what their anatomy is and exactly what's going on and … what surgeries and when. … And sometimes the notes aren't always consistent. … something will be called a double outlet right ventricle somewhere, but be called something completely different somewhere else and, depending on how sick a kid is, a very short amount of time, you're either delegating that to a trainee to try to get you the information, trying to get something from the parents, at the same time make decisions very, very quickly. … we often will we'll call cardiology,... But I think sometimes our sense of urgency is not always shared. … it's hard when we have the patient in front of us and they're getting worse.” (PEM02)*
Managing Time	Anticipate how long activities take and how timing affects patient care. *Example: “I felt the transition to the cardiac ICU would have potentially just delayed what he needed” (EXP03)*	Are mindful of time and focus on timely assessment of patient and their more definitive disposition. *Example: “It would be prudent to assess them quickly and start them on a pathway quickly, so they don't decompensate.” (PEM03)*
Team monitoring and management	Emphasize the importance of establishing a shared mental model with the broader team and engaging them in understanding the why (what is causing each symptom) as well as the what (the presenting symptoms). Monitor and ensure effective teamwork. *Example: “When you explain the why people pay more attention. So, I usually explain the why, because they grasp the concept better and then your concern becomes their concern” (EXP04)*	Emphasize the importance of establishing a shared mental model with the broader team with a focus on understanding the what (presenting symptoms and overall state) and the necessary actions (what tests to send and timing/priority). *Example: “I always try and verbalize what my thought is out loud to the room. So, you know, to kind of take control of the room, say, listen, we've got this two-year-old kid with known unrepaired Tetralogy …” (PEM06)*
Self-awareness and seeking consultation	Appreciate the risk associated with interventions and physiological states. Take steps to mitigate risk when possible. Believe that certainty is difficult to achieve, its most important to ensure decision making is not impeded by uncertainty. *Example: “I think having a sense of confidence and optimism does not imply a sense of certainty. It enables you to be uncertain and it just heightens your level of evaluation. Continuous evaluation and trying to minimize that uncertainty.” (EXP02)*	Appreciate the CHD population as a high-risk population overall and are aware and cognizant of their knowledge gaps pertaining to the population. Also acknowledge that decision making should not be impeded by uncertainty. *Example: “(in) ED, … uncertainty and how we manage it is very big in our practice. We have to be comfortable with what we don't know, but what we do know we can work with it.” (PEM07)*

In the following sections, the differences between CHD experts and ED physicians for *sensemaking, anticipation*, and *managing clinical complexity* will be described in greater detail as these were the most expressed and differed macrocognitive dimensions in the study.

### Sensemaking

Both CHD experts and ED physicians described using patients' clinical presentation, history, and data from the electronic health records (EHR) as their source of information and data for sensemaking. Both emphasized the importance of a physical exam and the utility of additional diagnostic tests to refine a diagnosis and verify a hypothesis or effectiveness of treatment. They both also visualized the cardiac anatomy to understand patients' presentation, although ED physicians described difficulties visualizing the CHD defects with the cardiac or surgical names alone and reported using other sources to help them visualize the anatomy. One CHD expert, for instance, described “*I kind of close my eyes and I imagine the heart that they are telling me about in the handover as a visual image and then from there I conclude my physiology”* (EXP03), while an ED physician said “*I have trouble visualizing, especially post-op kids and other forms of congenital heart disease. Those are more difficult for me”* (PEM04). In fact, CHD experts described patients' cardiac condition in detail including the functional of state the heart (e.g., how well the heart muscle was contracting and overall cardiac output) and amount of blood flow through the lungs, as well as describing the underlying cardiac anatomy. EXP02, for example, describes their thought process in understanding their desaturated (low oxygen saturations in the blood) single ventricle patient as follows: “*the main problem is just not enough oxygen getting into the bloodstream to be distributed to the rest of the body. And that is because of either not enough blood flow through the lungs to pick up oxygen, or the blood that is flowing into the lungs is actually being bypassed, or because the lungs are injured, that effective exchange doesn't occur. So it's either a problem with blood flow in the lungs or downstream. … the problem with that is you don't get enough blood flow through the lungs back to the heart, which can in turn affect its ability to pump blood to the circulation. But more so, it's the actual amount of oxygen in the blood is decreased because of both. (An intercurrent viral illness compromises gas exchange and treating it with positive pressure ventilation compromises blood flow to the lungs which is made worse given the immature lung vessels of the patient with this Glenn circulation).”* ED physicians, on the other hand, described their patients' cardiac condition using the overarching diagnosis of the CHD and the stage of the repair the patient may have had, but not in terms of the function state of the heart or implications of the underlying cardiac diagnosis. For instance, PEM03 described their thought process in treating their desaturated single ventricle patient as follows: “*(patient's) pathology is hypoplastic left heart. They were repaired. So their baseline oxygen saturations were typically in the 90's. But we accepted a little bit lower because of their chronic lung disease as well. … they had come in with desaturations to the 80's which was a bit unusual for her. … in the context of her having an upper respiratory illness … and on top of that had had some issues with fluid overload in the past. … we gave her a 10 cc per kilo bolus because she was a bit dry*.”

CHD experts were found to use the EHR system for relevant data to aid in the synthesis of differential diagnoses and need for additional investigations and interventions. However, ED physicians expressed that cardiology notes can at times be “*very convoluted with many cardiology terms that maybe don't make sense to us*” (PEM05). Patients' state was described by the CHD experts in reference to what they anticipated or deduced from the available data of the anatomy and function of various cardiac structures. For example, while providing advice over the phone to a peripheral hospital, EXP05 describes: “*because it's a right dominance, AV canal (Atrioventricular septal defect where the size of the right ventricle and its contribution to overall circulation is greater than the left ventricle given that there is no separation between the right and left side of the pumping chambers of the heart and its valves), certainly for the saturations, there is a degree of mixing and saturations will be lower (in the 80's), especially focusing on the preductal saturations. That would have had antegrade flow. How much desaturation would to some extent depend on how dominant (the right ventricle is) and where the flows are.”* In contrast, and without knowledge of the expected trajectory of a patient with a specific CHD, ED physicians relied on descriptions from parents and caregivers as to the baseline condition of their child. ED physicians expressed that obtaining baseline information from parents was difficult when there was a language barrier and when the patients' anatomy was complex. Similarly, obtaining information from the EHR was described by ED physicians to be difficult due to limited access (e.g., during patient resuscitation), complex language, and the variety of references within the EHR. When describing their interventions, CHD experts described how they manipulated specific aspects of cardiac physiology to stabilize the patient. For example, in a patient who had suffered a traumatic brain injury but also had a severe form of CHD and had previously undergone a highly specialized surgical procedure (called the modified Fontan operation), EXP07 described using norepinephrine to increase the blood pressure and perfusion to the brain without “*impeding (the patient's) cardiac function, which would worsen his Fontan pressures and worsen the cerebral edema.”* ED physicians described targeting their interventions toward restoring a previous state of stability. For example, PEM03 describes giving “*her some fluid because she wasn't drinking as well.”* When discussing fluid management in these patients, all ED physicians were very cognizant of the fluid sensitive state of these patients describing that they would be “*judicious”* and “*not wanting to fluid overload”* these patients. Finally, CHD experts very quickly focused on a cardiac diagnosis or support of the cardiac system while treating other etiologies. ED physicians, on the other hand considered all possible diagnoses before committing to a cardiac diagnosis. Of note, both CHD experts and ED physicians explored a wide range of possible causes for a patient's presentation, the CHD experts, however, focused on investigating a cardiac cause and supporting it as a priority. One exception to this finding was the rapid recognition of a “hypercyanotic spell” in a patient with a particular and known CHD called Tetralogy of Fallot (during which the patient became very cyanosed or blue from a very low oxygen level); in this circumstance the ED physician described initiating urgent treatment while at the same time working on ruling out other differential diagnoses. EXP04, described that regardless of presentation, it's important, in this population, to “*see everything from the perspective of the heart”* while ED physicians emphasized that when a patient presents, it was important to keep an open framework and to not “*turn off your thinking with cognitive biases”* (PEM04). As such, CHD experts spent more time describing why the patient was presenting as they were, while the ED physicians focused on detecting what was going on with the patient.

### Anticipation and Managing Clinical Complexity

Given that CHD experts quickly narrowed in on a cardiac cause for a patient's presenting symptoms, they spent much of their time discussing how they anticipated the patient's trajectory evolving and how they planned on mitigating potential problems. This was in contrast to ED physicians who maintained a broader differential for a longer period of time, particularly given the vague symptomatology of heart failure in these patients. As such, they devoted much of their attention to managing the complexities of the influx of data prior to arriving at a diagnosis. This includes searching for a patient's baseline vital signs either in the electronic health records or in conversations with care providers to establish an understanding of patient acuity and risk or using POCUS to determine the safety of fluid administration. This contrasted with CHD experts who knew what vital signs to expect of the patient as their baseline and the likelihood of a CHD tolerating a volume load based on the patient's CHD diagnosis and without POCUS. This allowed CHD experts to pursue a proactive approach to patient management while ED physicians were reactive to the data they would obtain. Thus, CHD experts anticipated how a situation might unfold by categorizing their patients and interventions in risk groups based on patients' specific cardiac physiology, whereas ED physicians kept an open framework and categorized their patients in broad risk categories to allow for confirmation of disease based on incoming evidence.

While ED physicians did not describe anticipating a trajectory that was as specific as CHD experts, they reported anticipating that pediatric CHD patients who present with respiratory symptoms may require particular treatments such as fluid resuscitation, and therefore planned for additional sources of data, such as point of care ultrasound (POCUS) to inform their course of action. The anticipation and planning vary also on the complexity of the CHD and the anticipation of complications related to treatment or interventions. For example, consider a pediatric patient with cardiomyopathy in need of mechanical ventilation given a presentation of respiratory distress. In anticipating cardiac arrest during intubation, a CHD expert (EXP04) would identify that this could be “*secondary to changes in cardiac preload”* and afterload at the time of intubation and therefore initiate therapy that would support the heart in specific ways to minimize the chances of cardiac arrest. ED physicians, on the other hand, may not be able to anticipate the specific mechanism of cardiac arrest in these patients, but they anticipate the possibility of arrest during intubation and seek additional resources and expertise to similarly minimize risk. PEM06, for instance, explains closely consulting the CHD experts for some of these fragile CHD patients and considering “*is this someone that if you want to intubate them, when you do it in the unit with the surgeon next to you in case they arrest? (…) because I know that if this kid arrests doing compressions, (…) we are going to have a very hard time getting him or her back.”* In sum, while CHD experts spent more time anticipating course of action specific to CHD trajectory, ED physicians spent more time managing complexities associated with determining the correct diagnosis and anticipating specific courses of actions that may be required based on the eventual confirmed diagnosis.

## Discussion

The present findings contrast the strategies of CHD experts and ED physicians when treating CHD patients. Our findings show that ED physicians lack a mental model for visualizing patterns in the CHD defects based on the cardiac or surgical names alone and reported needing other sources to help them visualize the anatomy. Consequently, ED physicians were forced into a reactive mode (i.e., they spend a prolonged time gathering a wide range of data and gradually initiating therapies and reacting dynamically to what they encounter) as opposed to a proactive stance to anticipate CHD specific problems and preventing them. Specifically, our findings highlight that ED physicians require help with visualizing structural heart defects in CHD patients, especially those involving multiple defects and lesions of the heart and its great vessels. This was particularly difficult when ED physicians had to rely on written descriptions of these complex heart defects in echocardiography reports, cardiology notes, and on parental descriptions. There is a pressing need to determine how best to illustrate these complex CHDs using graphics and to make clinically relevant data readily available to clinicians to facilitate sensemaking and patient care. This is very useful information for system designers to help them understand the needs of the ED physician. Thus, the study findings can help inform the design of solutions such as decision-support systems, or to revise the way ED physicians are trained to treat CHD patients.

In their study, Schubert and colleagues had compared the differences in macrocognitive processes between expert and novice emergency medicine physicians (15). They found that compared to novices, expert ED physicians maintained an open framework when diagnosing patients presenting to the ED (15). Experts were also open to incorporating new data into their top differentials in an attempt to refine their diagnosis to what most accurately reflects the patient's condition (15). Similarly, the ED physicians from our study maintained a broad differential and gathered data to refine their diagnosis before coming to a cardiac cause and diagnosis for the presentation of pediatric CHD patients. This was in contrast to CHD experts who recognized a cardiac cause to the presentation and started targeted interventions while at the same time gathering additional information to definitively rule out other illness etiologies. This faster recognition of cardiovascular compromise led to CHD experts spending more time anticipating their targeted interventions and preventative measures to avoid further patient deterioration. As such, our results suggest that keeping an open framework, which is considered necessary in the ED overall, can delay the initiation of targeted therapies for CHD patients which may affect outcomes. We therefore suggest interventions that improve the efficiency of ED physicians' sensemaking with respect to CHD patients so that they could move into anticipating CHD specific trajectories.

One way to support non-experts in CHD, such as the ED physicians in this study, is the development of readily accessible clinical decision support systems and protocols that enable relevant patient-specific data and the corresponding CHD specific *frames* to be available. *Frames* are perspectives that shape and define the available data and dynamically change in response to existing or new data (29, 30). These frames are central to the process of sensemaking which is the process of understanding a current state (30, 31). Sensemaking can occur retrospectively and prospectively. Retrospective sensemaking, or understanding a state based on previous or existing data, is most relevant to the process of making a diagnosis. In this process clinicians create frames based on presenting symptoms and patient history to develop an overarching diagnosis. The ED physicians in our study sought out historic information from the EHR and the patients' parents to understand their CHD and the context of their current presentation before arriving at a diagnosis. Similarly, in looking through cardiology notes of patients, ED physicians searched for existing, patient specific, frames of CHD experts to facilitate their own framing and retrospective sensemaking of patients' current state. Prospective sensemaking refers to sensemaking where the focus is primarily toward events that may occur in the future (32). In the operating room, prospective sensemaking was found to enable the operating team to construct plausible projections of what might happen and how they would act should such futures occur (32). They found that prospective sensemaking allowed these clinicians to plan for contingencies, work through decision dilemmas, and structure the future course of the patient through their current actions (32). Effectively, prospective sensemaking allows for the creation of mental maps that complement the frames created as a result of retrospective sensemaking. CHD experts in our study also applied prospective sensemaking to predict the course of CHD patients based on their specific CHD diagnosis, historical and presenting data, as well as their current state. Sharing patient specific CHD frames and ways to test their relevance in the context of a patient allows ED physicians to prioritize investigating a cardiac cause for the presentation, focus on how they manage clinical complexity and data flow while maintaining an open frame. Facilitating prospective sensemaking in this regard can shift ED physicians toward CHD specific anticipated actions.

### Influencing Clinician Macrocognition and Decision Making

In addition to facilitating a greater understanding of clinical expertise and decision making, macrocognitive methods have also been used in cognitive training (33), clinical decision support systems (CDSS) (16, 20, 21), and automation (34, 35). Additional training in the field of pediatric CHD was deemed ineffective by participants in Cashen et al.'s (9) study due to the relative infrequency in the presentation of these patients to community EDs. Furthermore, the differences in macrocognition between ED physicians and CHD experts in our study were appropriately suitable to their clinical role and work environment. ED physicians in our study indeed commented on potential risks associated with changing their macrocognition to match that of other subspecialty experts such as CHD experts. In this context, therefore, cognitive training to change the macrocognition of ED physicians to match that of CHD experts would not be an optimal approach to augmenting their decision making as it relates to CHD patients.

Incorporating ED and CHD expert macrocognition in the design of a patient specific, easily accessible, and up to date CDSS could also augment clinician decision making as it relates to the acute care of pediatric CHD patients. Emergency department physicians in our study described referring to the EHR and speaking to parents to obtain data on baseline physiological measures of the patient (e.g., saturations, heart rate, and blood pressure) and the patient's cardiac CHD diagnosis. These physicians indicated having difficulties visualizing the CHD and described looking for patients' latest echocardiography as well as medication list to understand patients' presentation. Given that this information is central to clinicians' retrospective sensemaking, they could potentially be incorporated in the design of a CDSS. To facilitate prospective sensemaking and anticipation, a CDSS could also provide ED physicians with the information that CHD experts deliberate before proceeding with taking anticipatory steps. These can include considerations to resuscitation of the patient and potential challenges associated to patients' successful rescue as well as information on the physiology of a particular CHD, how they would respond to various interventions, and what interventions should be considered for a given physiology. The data for such a tool could be obtained from patients' charts and approved by their cardiologist before becoming available on mobile devices of patients or parents. Where possible, it could also be integrated into existing EHRs to further facilitate clinician accessibility to the tool and its contents. A graphical depiction of what such a tool could look like can be found in Supplementary Figure 1.

Regardless of how macrocognition is used to influence clinical decision making, all solutions should be evaluated using various human factors techniques such as heuristics and usability testing of any electronic solution, including CDSS.

## Limitations

This was a single center study which made the sample size small with a possible effect on the generalizability of the findings to other centers and roles. Given that the study site was a major cardiac center, the ED physicians in this study not only saw CHD patients more frequently than those in a non-cardiac center, but they also had access to existing protocols and frameworks that were implemented to streamline and expedite access to CHD experts when CHD patients present acutely. The decision making of these ED physicians may have also been affected by these frameworks which may not exist elsewhere. Nevertheless, it is expected that ED physicians in non-specialty centers would have similar but more pronounced findings as those found here. There is also a degree of recall bias as participants relied on their memory to remember a scenario, their interventions, and decision-making at the time. We don't believe this is a significant limitation as the focus of the study has been on the decision-making processes rather than the specific sequence of actions taken to treat the patient.

## Future Directions

This study will be expanded to include participants from another institution to verify findings with a plan to further expand to include adult patients with CHD and their adult ED physicians.

## Conclusion

In comparing the macrocognitive processes of CHD experts and ED physicians, this study highlights some key differences in their decision-making pertaining to acutely ill pediatric CHD patients. Specifically, this relates to the macrocognitive processes of *sensemaking* and *anticipation* which can be explained by the differences in framing, data processing, and access to frames which develop with experience and specialized training. CHD experts were found to utilize their experience and CHD specific knowledge to determine their expectations of physiological measures as well as potential CHD related differentials for the acute presentation for any given CHD. As a result, their sensemaking was more detailed and their anticipatory actions and considerations were more specific compared to ED physicians. Conversely, sensemaking among ED physicians was highly dependent on the data they could gather from a patient and parents as well as the EHR. They also appreciated pediatric CHD patients to have a fragile hemodynamic and anticipated their deterioration broadly but relied on the CHD experts to delineate the mechanism of deterioration and appropriate course of action. These findings should be incorporated into the design of sociotechnological solutions such as clinical decision support systems, to augment clinician *sensemaking and anticipation* while also improving access to information overall.

## Data Availability Statement

The raw data supporting the conclusions of this article will be made available by the authors, without undue reservation.

## Ethics Statement

The studies involving human participants were reviewed and approved by Research Ethics Board of the Hospital for Sick Children. The patients/participants provided their written informed consent to participate in this study.

## Author Contributions

AA analyzed and interpreted the data as well as conduct the study and drafted the initial manuscript. PL and PT contributed to the data interpretation and the study design as well as subsequent editing of the manuscript. PL provided clinical insight while, PT provided expertise on study method and principles of cognitive task analysis. GF facilitated with protocol refinement and added valuable insights from the ED when interpreting the data. All authors contributed to the article and approved the submitted version.

## Funding

This project, as part of a PhD studentship was funded by the Natural Sciences and Engineering Research Council of Canada (NSERC: CRE-ATE) and SickKids' Heart Center Innovation Fund.

## Conflict of Interest

The authors declare that the research was conducted in the absence of any commercial or financial relationships that could be construed as a potential conflict of interest.

## Publisher's Note

All claims expressed in this article are solely those of the authors and do not necessarily represent those of their affiliated organizations, or those of the publisher, the editors and the reviewers. Any product that may be evaluated in this article, or claim that may be made by its manufacturer, is not guaranteed or endorsed by the publisher.
