# Supplementary material for: Understanding Clinician Macrocognition to Inform the Design of a Congenital Heart Disease Clinical Decision Support System
Source: Front Cardiovasc Med. 2022 Feb 3;9:767378. doi: 10.3389/fcvm.2022.767378 (PMC8850471; doi:10.3389/fcvm.2022.767378)
Supplement: Supplementary file 1 [file Image_1.PDF]

Supplementary Material

Basic Demographics

My Resuscitation Status is **No E-CPR**

I have a Grade 1 airway

I Weigh 4.00 Kg

I am Allergic to:
penicillin

B

My Saturations Range:
70-80

My Heart Rate Range:
130-140

My MBP Range:
45-50

My Cardiac Anatomy is:
Hypoplastic Left Heart Syndrome post Norwood-BTS operation

A

This is What My Heart Looks Like

A

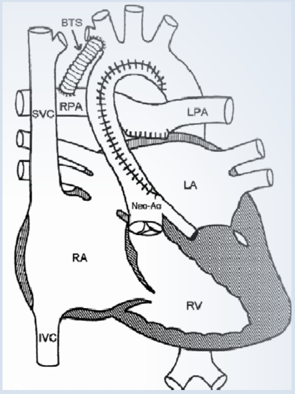

When Acutely Ill

My Latest Lead II ECG

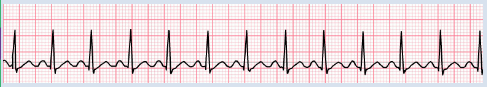

History of Arrhythmias:
AET, Atrial Flutter

Permanent Pacing:
No

My Latest Echocardiography showed:
Trivial RAVVR
No AI
Unobstructed flow across the Ao arch
Patent BT shunt
Laminar flow across the PAs
Mildly depressed ventricular function

A

Occluded Vessels:
RIJV - completely occluded RICA - completely occluded

B

Current relevant Medications:
Furosemide 3.5mg BID PO; ASA 20.25mg OD PO

A

Important Comorbidites:
LMCA stroke

Physiological Considerations

B

Single Ventricle: Norwood BTS

*I only have one ventricle that pumps blood to both my lungs and my body
*I am anticoagulated for a shunt that provides blood flow to my lungs from my innominate artery
*If my saturations are >85%, then my organs may not get enough blood and too much may go to my lungs

- My Heart can't handle too much volume
- Make it easier for blood to flow to my body
- Help my heart squeeze better
- Decrease the demand on my heart

*If my saturations are low, my shunt may be clotted:

- My saturations would be UNRESPONSIVE to supplemental O2, rapidly leading to hypoxia induced bradycardia and PEA
- You may not be able to hear my shunt murmur in the L/RUSB
- If you are monitoring my end tidal CO2, it would be reduced

C

- Treat with:
  - Administration of 100% O2
  - Vasoconstrictive/inotropic support: prearrest dose of epinephrine (1mcg/kg/dose) to increase blood pressure and flow across the shunt
  - Anticoagulation (50-100U/Kg heparin)
  - Intubation
  - I may need ECMO support and URGENT Cath/Surgery

In Case of Cardiac Arrest

1. Pre-arrest phase: CRITICAL
- Consider giving Pre-arrest dose epi - 1mcg / kg / dose

2. Arrest Phase:
- Follow PALS recommendations with potential modifications based on the unique functional state
- Compressions 100-120/min with full recoil (4-5cm in infants and children)
- Arrest dose epi is 10mcg/kg/dose
- High quality CPR is vital in improving chances of ROSC
- Delivery of high quality CPR may be impaired by underlying structural heart disease

3. Post-arrest Phase:
- Immediate assessment of cardiac recovery

C

Successful Resuscitation May be Limited by:

B

⊟ Limited Stroke Volume:

From Impaired EF

⊟ Limited Pulmonary Blood Flow:

Elevated PVR

** Passport Approved by staffMD @ 2021-09-26 10:37:06 AM **

Hospital Doctors, if you are worried about me and would like some help looking after me, Please Activate CritiCall Ontario (1-800-668-4357) for Help and Possible Transfer

C

Supplemental Figure 1 - A potential example of a CDSS designed with considerations to macrocognitive processes of CHD experts and ED physicians. *Section A* depicts information facilitating retrospective sensemaking. *Section B* depicts information facilitating prospective sensemaking. *Section C* depicts information facilitating anticipation.
